# Gut microbiota of homologous Chinese soft-shell turtles (*Pelodiscus sinensis*) in different habitats

**DOI:** 10.1186/s12866-021-02209-y

**Published:** 2021-05-11

**Authors:** Benli Wu, Long Huang, Jing Chen, Ye Zhang, Jun Wang, Jixiang He

**Affiliations:** 1 Key Laboratory of Aquaculture & Stock Enhancement of Anhui Province, Fisheries Research Institute, Anhui Academy of Agricultural Sciences, No.40 Nongkenan Road, Luyang District, Hefei, 230031 Anhui Province China; 2 State Key Laboratory of Eco-hydraulic in Northwest Arid Region of China, Xi’an University of Technology, 710048 Xi’an, China

**Keywords:** Gut microbial variation, Diversity, Habitat, Rice-turtle coculture.

## Abstract

**Background:**

Chinese soft-shell turtle (*Pelodiscus sinensis*) is an important commercial species for their high nutritional value and unique taste, but it has been a vulnerable species due to habitat loss. In this study, homologous juvenile turtles were allocated to lake, pond and paddy field to investigate the habitat effects on turtles.

**Results:**

The growth, morphology and gut microbial communities were monitored during the 4 months cultural period. It showed higher growth rate of turtles in paddy field and pond. The appearance, visceral coefficients, gut morphology and microbial communities in turtles were distinct among different habitats. The microbial community richness on Chao1 was obviously lower in initial turtle guts from greenhouses, whereas it was relative higher in turtle guts sampled from paddy fields than ponds and lake. Significant differences on dominant microbes were found among initial and subsequent samples from different habitats. Firmicutes was the most abundant phylum in the guts of turtles sampled from the greenhouse initially, while Proteobacteria was the most abundant phylum after cultivation in different habitats, followed by Bacteroidetes. The microbial composition were distinct in different habitats at 60d, and the appearance of dominant phyla and genera was more driven by sampling time than habitats at 120d. Both the sampling time and habitats affected the appearance of dominant phyla and genera during the cultivation. The functional predictions indicated that both habitat type and sampling time had significant effects on metabolic pathways, especially amino acid and carbohydrate metabolism.

**Conclusions:**

The turtles could adapt to natural lakes, artificial ponds and paddy fields. The gut microbial abundance was different among the habitats and sampling time. The species of microbes were significantly more diverse in paddy field specimens than in those from ponds and lakes. Rice-turtle coculture is a potential ecological and economic farming mode that plays important roles in wild turtle protection and food security.

**Supplementary Information:**

The online version contains supplementary material available at 10.1186/s12866-021-02209-y.

## Background

Owing to their high nutritional value and unique taste, the consumption of wild animals is popular in many countries and areas, which has accelerated the recession of wild resources. However, some of the wild animals that are consumed may carry highly pathogenic viruses and bacteria that pose a potential threat to humans. Moreover, wildlife conservation has become an impending issue in recent decades. Chinese soft-shell turtle (*Pelodiscus sinensis*, hereinafter referred to as turtle) is an important commercial aquatic species in Southeast Asia, including China, and is commonly considered a tonic food due to its high nutritive and medicinal value [1, 2]. *P. sinensis* has become a vulnerable species due to habitat loss and overfishing [3]. These turtles live in water and have been traditionally cultured in lakes, rivers or reservoirs at low stocking densities. Currently, several modes of captive culture have been promoted to satisfy market demand; greenhouses and artificial ponds are commonly selected as sites for high production turtle aquaculture [4]. Intensive cultivation can significantly increase the production of commercial turtles while shortening the culture period; however, it is also accompanied by problems such as a high risk of disease, defective appearance and low quality. Furthermore, the high energy consumption of these rearing patterns is not conducive to sustainable agricultural development and has aroused increasing public concern [4, 5]. Intensive breeding can cause diseases such as bacterial infection and mesenteritis in aquaculture species due to the associated crowded living spaces and superfluous but simple food sources [6–8]. Consequently, antiseptic medicines and antibiotics have been abused leading the rise in antibiotic resistance [9, 10], thus leading to more serious environmental and health problems that affect both the quality of the product and animal welfare [11–13]. Probiotics have been developed for both cultured animals and humans as immunopotentiators [14, 15], but their positive effects are limited and temporary; thus, more healthy culture modes should be applied for high-quality aquatic products [16, 17].

Turtles from different habitats generally show obvious differences in appearance, morphology, textural properties, chemical composition and flavor substance contents [5, 18]. Physiological disorders are associated with marked changes in gut microbial communities. The gut microbiota is a protective barrier of organisms to prevent pathogen invasion and is affected by both the internal and external environment for mammals as humans and mouses [19–21]. The diversity and variation of gut microbial communities have been considered indicators of the health status of cultured fishes [22]. In recent years, the coculture of rice and aquatic animals, such as rice-fish, rice-crayfish and rice-turtle systems, has been rapidly developed in Southeast Asian regions, especially in South China [23]. Paddy fields can provide capacious space, shelter and natural food for cultured animals. Cultured animals prey on pests, and the activities of the cultured animals could loosen the soil and provide organic fertilizer for paddies, thus significantly decreasing the utilization of chemical fertilizers and pesticides [24, 25]. Therefore, the coculture mode has been considered an economic and ecological culture mode in rice-growing regions.

Host genetics, diets and ambient environmental conditions could affect the composition of the complex gut microbiota in fish [26, 27]. But it is difficult to fully unravel the diversity and dynamics of gut microbiota and identify keystone species for specific functions [16]. In the present study, homologous juvenile turtles with similar genotypes and early life conditions were allocated to different habitats to investigate their differences in growth and morphology and analyze the diversity and variation in their gut microbial communities within cultural periods. Efforts were also made to identify functional microbes or representative communities as biomarkers of the physiological status of turtles in different habitats.

## Results

### Turtle growth and morphology under different habitats

Mortality was negligible in both paddy fields and ponds during the experimental period. However, only a small number of turtles were caught from the lake at 60 d, and no marked turtles were recaptured at 120 d, resulting in incomplete statistics for mortality and growth for turtles in the lake. No wild turtles were caught during sampling. A relatively small sample size (n = 3) for each group was designed for turtle resource protection purposes, and it was difficult to sample from natural lakes. There were significant differences in growth among the different groups; the body weights of turtles in paddy fields and ponds were obviously higher than those in lakes (*p* < 0.05), and divergence occurred in the early days. The growth rates of turtles were 0.76 %/d, 0.68 %/d and 0.40 %/d for those from paddy fields, ponds and lakes, respectively, in the first 60 d. The rate was 0.72 %/d and 0.62 %/d for turtles from paddy fields and ponds, respectively, during the whole 120 d. The hepato-somatic index and clumpy fat index were highest in ponds, second in paddy fields and lowest in lakes (*p* < 0.05). The gut-somatic index of weight (DSI_W_) for turtles from ponds was significantly higher than that for turtles from lakes and paddy fields (*p* < 0.05). Conversely, the gut-somatic index of length (DSI_L_) was higher for turtles from paddy fields and lakes than those from ponds. Measured values are presented as the mean ± standard deviation, and the different superscript letters in the same row indicate significant differences (*p* < 0.05) **(**Table 1**)**.

**Table 1:** The anatomical indices of turtles from different habitats and cultured days

Indices	0d			60d			120d	
	Field	Pond	Lake	Field	Pond	Lake	Field	Pond
BW	335.8 ± 22.2	341.3 ± 32.6	344.9 ± 26.8	529.7 ± 35.5^b^	512.2 ± 39.3^b^	438.8 ± 27.4^a^	796.6 ± 58.2^b^	717.2 ± 64.6^a^
CL	12.89 ± 0.09	12.91 ± 0.06	12.92 ± 0.07	15.96 ± 0.26^b^	15.87 ± 0.30^b^	15.30 ± 0.28 ^a^	17.71 ± 0.35^b^	17.13 ± 0.38^a^
CW/CL	0.909 ± 0.006	0.914 ± 0.002	0.914 ± 0.003	0.768 ± 0.006^a^	0.766 ± 0.08^a^	0.779 ± 0.005^b^	0.782 ± 0.010	0.786 ± 0.008
CLW/CL	0.150 ± 0.002	0.149 ± 0.002	0.149 ± 0.002	0.164 ± 0.006^a^	0.178 ± 0.005^b^	0.174 ± 0.008^b^	0.203 ± 0.06^a^	0.217 ± 0.010^b^
SGR	-	-	-	0.76	0.68	0.40	0.72	0.62
HSI	2.9 ± 0.2	2.9 ± 0.2	2.9 ± 0.2	3.0 ± 0.3^b^	3.1 ± 0.2^b^	2.8 ± 0.3^a^	2.6 ± 0.2^a^	2.7 ± 0.2^b^
FSI	3.8 ± 0.2	3.8 ± 0.2	3.8 ± 0.2	3.6 ± 0.2^b^	4.2 ± 0.2^c^	2.9 ± 0.2^a^	3.6 ± 0.1^a^	3.9 ± 0.2^b^
GSI_W_	2.6 ± 0.1	2.6 ± 0.1	2.6 ± 0.1	2.1 ± 0.1^a^	2.3 ± 0.1^b^	2.1 ± 0.1^a^	2.1 ± 0.0^a^	2.4 ± 0.1^b^
GSI_L_	4.0 ± 0.2	4.0 ± 0.2	4.0 ± 0.2	4.0 ± 0.1	3.9 ± 0.1	4.0 ± 0.1	3.7 ± 0.2^b^	3.5 ± 0.2^a^

BW (g): body weight

CL (cm): carapace length

CW (cm): carapace width

CLW (cm): calipash lateral width

SGR (Specific Growth Rate,%/d):100×(Ln(BW_T_)-Ln(BW_0_))/T

HSI (Hepatosmatic Index,%) = 100×liver weight / BW

FSI (Clumpy Fat Index,%) = 100×clumpy fat weight/ BW

GSI_w_ (Gut-smatic Index on Weight,%) = 100×gut weight/BW

GSI_L_ (Gut-smatic Index on Length) = gut length/ Carapace length

There was no obvious trauma experienced by most turtles from the lake except occasional parasitic leeches observed on the calipash. However, more bruises or scars were observed for the turtles from ponds than those from paddy fields. The appearance, such as the color, of the carapace and plastron were different among turtles from different habitats. The carapace of turtles cultured in ponds presented a bottle green color, but the individuals from paddy fields presented a bottle green color with a slight golden yellow color, which was similar to turtles from lakes. There were significant differences in carapace width/carapace length (CW/CL) and calipash lateral width/carapace length (CLW/CL) values at 60 d and 120 d (*p* < 0.05), and the CW/CL value was relatively higher for turtles from lakes, and CLW/CL was higher for turtles from lakes and ponds than for those from paddy fields (*p* < 0.05) (Table 1).

### Composition and diversity of turtle gut microbiota

**Fig. 1:**
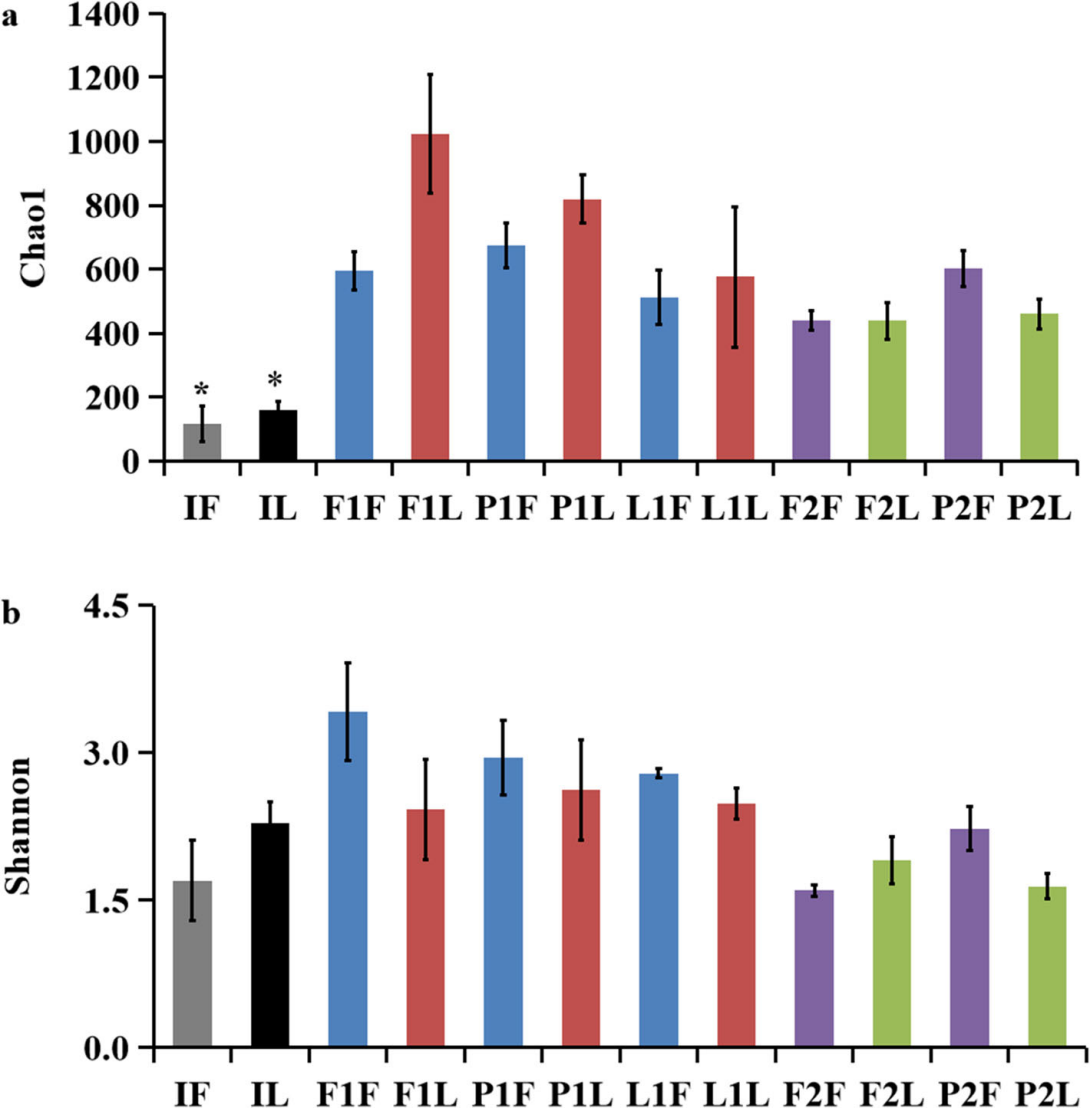
Richness (Chao1, a) and evenness (Shannon, b) indices of gastrointestinal microbial communities on OTUs, the *on the bar indicated significant differences with other groups, the grouping details were listed in Table 2

**Fig. 2:**
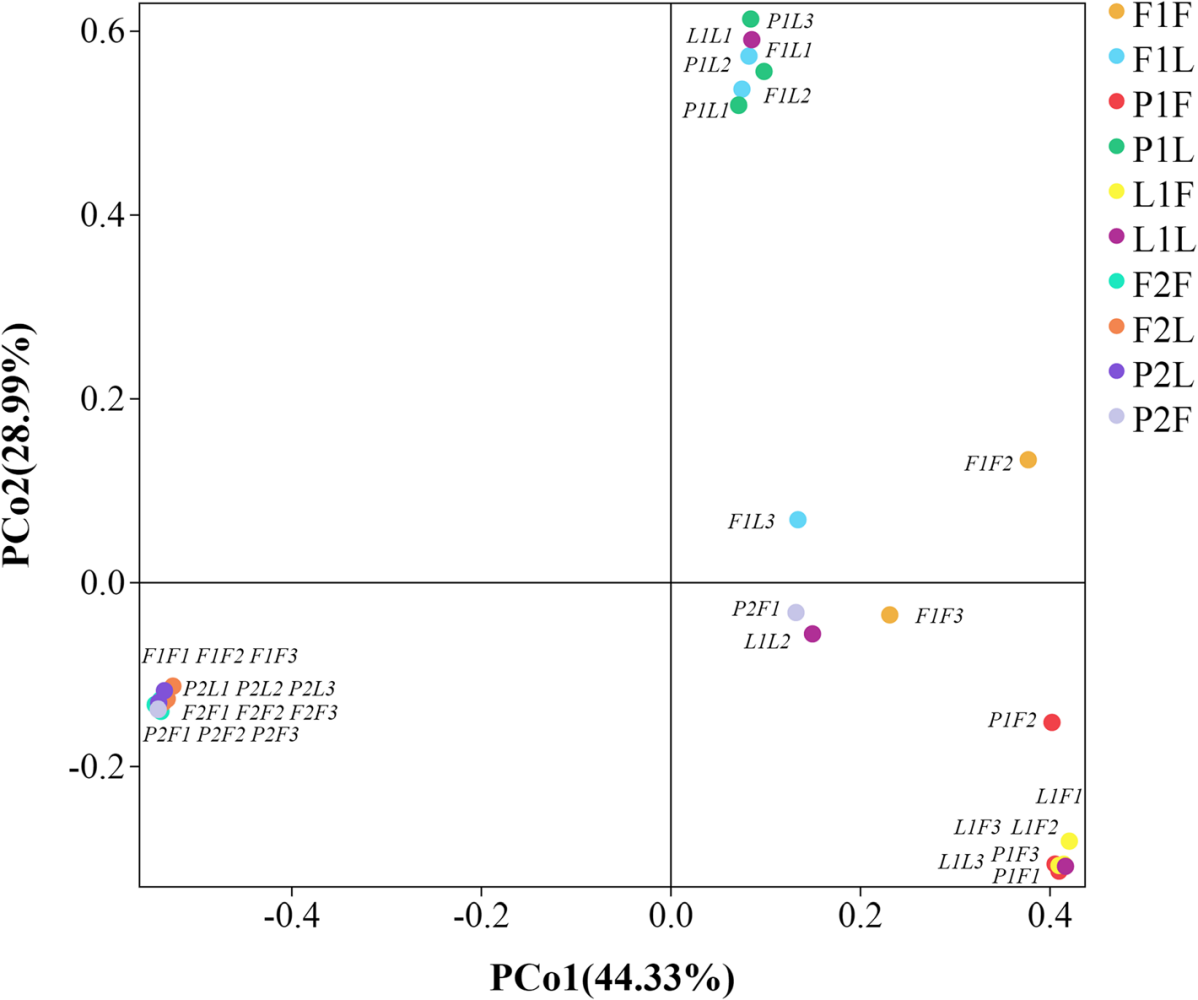
The PCoA (principal co-ordinates analysis) on Bray-Curtis, the different symbols represented different groups from different habitats and cultural periods, the grouping details were listed in Table 2

The grouping details for samples from different habitats, culture days and intestinal segments are listed in Table 2. For gut samples, a total of 1 723 158 valid bacterial 16 S rRNA gene reads were obtained, and 4 901 OTUs were identified from all samples. The observed total OTUs varied from 64 ~ 822. The total number of OTUs was significantly lower in initial groups IF and IL and higher in groups F1F and F1L from paddy fields at 60 d. The number of OTUs was 17 ~ 48, representing more than 0.01 % of the total OTUs (Table S1). Significant differences were found in OTU composition among groups (Fig. S1). Guts sampled at 120 d had few unique OTUs, both in the foregut and hindgut. Rarefied OTUs (with reads normalized to 35,000 for each sample) was adopted to do diversity and richness analysis. We picked Chao1 and Shannon as richness and evenness indicator, respectively. The index of Chao1 was higher in the hindgut than those in the foregut at 60d, whereas it was higher in turtle guts sampled from paddy fields than ponds and lakes. In addition, the Chao1 index was significantly lower in initial turtle guts from greenhouse compared to the samples from the three different habitats (*p* < 0.05) (Fig. 1a). The shannon index was relative higher in foregut than hindgut samples, whereas it was also relative higher in foregut samples from paddy field than pond and lake at 60d (Fig. 1b). The species and number of OTUs varied significantly at 60 d, different from the relatively similar results across groups obtained at 120 d. The microbial abundance was higher in samples from paddy fields than in samples from lakes and ponds during the experiment. The microbial community presented relatively high similarity in guts sampled at the same time. The PCoA (principal coordinate analysis) of the Bray-Curtis dissimilarity showed high microbial community similarity in guts from the same individual or group and significant discrepancy in samples from different habitats, sampling times and gut sections (Fig. 2, Fig. S2). Generally, both sampling time and habitat affected the variation in the gut microbial communities.

**Table 2:** Grouping details for samples from different habitats, cultured days and intestinal segment

Groups	Body weight(g)	Living habitats	Cultured days	Sampled gut segment
IF	340.5 ± 6.7	Greenhouse	0d (I)	Foregut (F)
IL		Greenhouse	0d (I)	Hindgut (L)
F1F	530.3 ± 5.6	Paddy Field(F)	60d(1)	Foregut (F)
F1L		Paddy Field(F)	60d(1)	Hindgut (L)
F2F	806.6 ± 10.2	Paddy Field(F)	120d(2)	Foregut (F)
F2L		Paddy Field(F)	120d(2)	Hindgut (L)
P1F	515.0 ± 7.3	Artificial Pond(P)	60d(1)	Foregut (F)
P1L		Artificial Pond(P)	60d(1)	Hindgut (L)
P2F	720.4 ± 3.3	Artificial Pond(P)	120d(2)	Foregut (F)
P2L		Artificial Pond(P)	120d(2)	Hindgut (L)
L1F	350.3 ± 5.1	Natural Lake(P)	60d(1)	Foregut (F)
L1L		Natural Lake(P)	60d(1)	Hindgut (L)

The letters or numbers in groups names indicated “Habitat”, “Sampling time"and “Gut segment”, respectively, which were also shown in the parentheses. Body weight here was average body weight of the three sampled turtles for each groups

### Dominant microbes

**Fig. 3:**
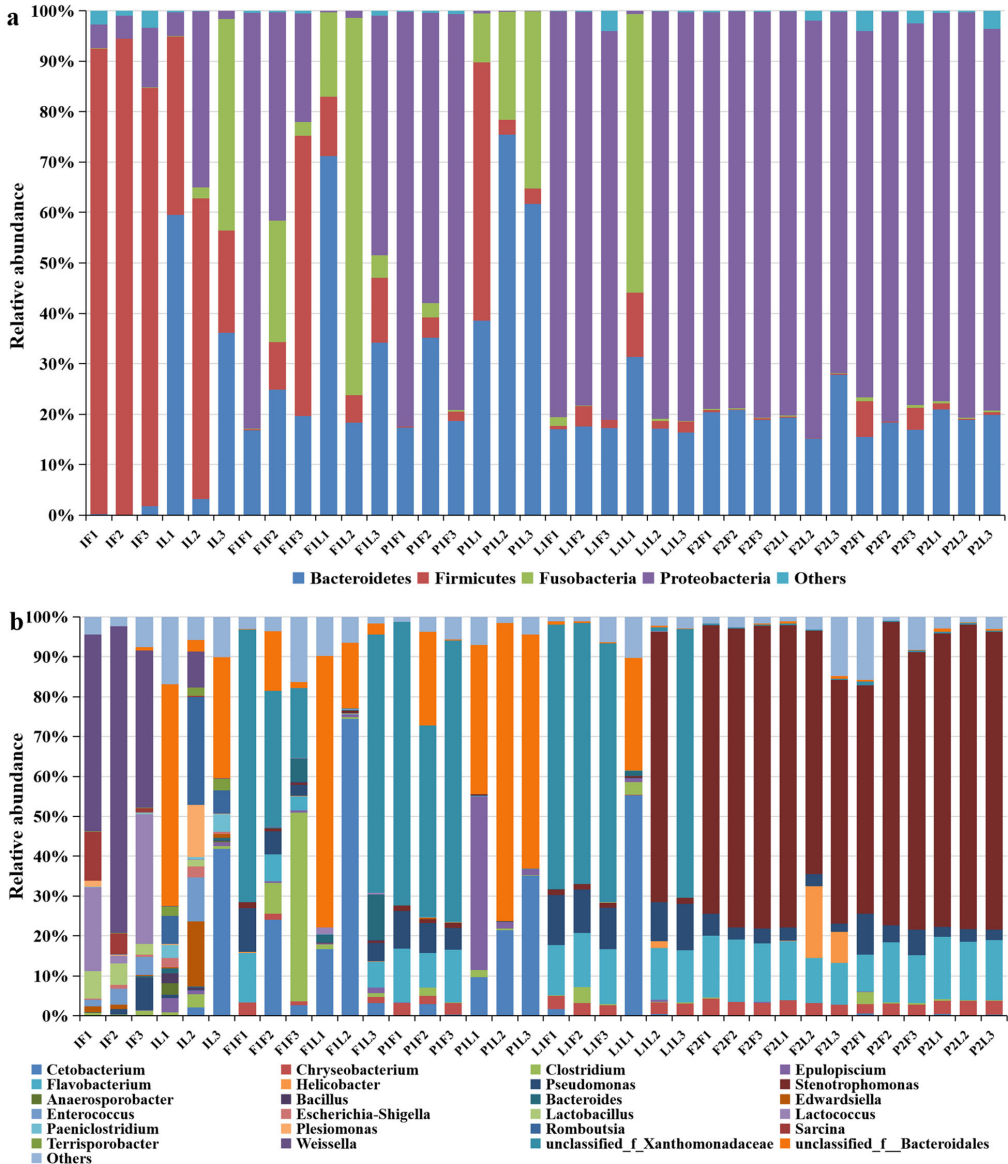
The dominant phyla (**a**) and genera (**b**) of gut microbial community under different habitats and sampling time, the grouping details were listed in Table 2 and the last number (1, 2, 3) in group label indicated sample number

The recognized microbes belonged to 27 phyla, 59 classes, 97 orders, 151 families, and 219 genera from all the samples based on GreenGene. The phylum and genus levels were emphasized in the analysis. Bacteroidetes, Firmicutes, Fusobacteria and Proteobacteria were the most dominant phyla, accounting for more than 95 % of the total bacteria in all samples. Firmicutes was the most abundant phylum in the guts of turtles sampled from the greenhouse initially, while Proteobacteria was the most abundant phylum after cultivation in different habitats, followed by Bacteroidetes. Firmicutes and Fusobacteria commonly existed at 60 d but were rarely present at 120 d in turtles from all three habitats (Fig. 3a). Additionally, the unidentified bacteria were more abundant in turtles from lakes than those from paddy fields and ponds.

There was a significant difference in dominant genera among initial samples and subsequent samples from different habitats. The dominant genera in the initial samples were an unclassified genus belonging to Bacteroidales, *Romboutsia*, *Cetobacterium*, *Weissella*, *Lactococcus*, *Lactobacillus*, *Clostridium*, *Edwardsiella*, *Plesiomonas*, and *Sarcina*. For samples from the three habitats mentioned above, the dominant genera were *Cetobacterium*, *Chryseobacterium, Clostridium*, *Epulopiscium*, *Flavobacterium*, *Helicobacter*, *Pseudomonas*, *Stenotrophomonas* and another unclassified genus belonging to Xanthomonadaceae. The abundance of dominant genera varied with habitat, sampling time and gut location. For turtles sampled from paddy fields, the most dominant genus in foregut samples taken at 60 d was *Clostridium*, and in the hindgut, it was *Cetobacterium*, while at 120 d, the most dominant genus was *Stenotrophomonas* both in the foregut and hindgut. For turtles sampled from ponds, the most dominant genera at 60 d were *Flavobacterium* and *Cetobacterium* in the foregut and hindgut, while at 120 d, the most dominant genus was also *Stenotrophomonas*. For turtles sampled from the lake, the most dominant genera at 60 d were *Flavobacterium* and *Cetobacterium* in the foregut and hindgut, respectively (Fig. 3b).

The dominant species in different gut locations were also distinct. In the foregut, the dominant species were *Weissella cibaria*, *Enterococcus durans*, *Lactobacillus sakei*, *Lactococcus lactis*, *Lactococcus garvieae*, *Sarcina sp.* and *Pseudomonas sp.*, whereas in the hindgut, *Clostridium sensu stricto*, *Romboutsia sp.*, *Weissella cibaria, Escherichia coli*, *Plesiomonas shigelloides*, *Edwardsiella tarda*, *Paeniclostridium sp.*, *Cetobacterium sp.*, *Terrisporobacter sp.* and two other unclassified species belonging to Bacteroidales were the most abundant.

### Microbial communities in turtles from different habitats and at different sampling times

**Fig. 4:**
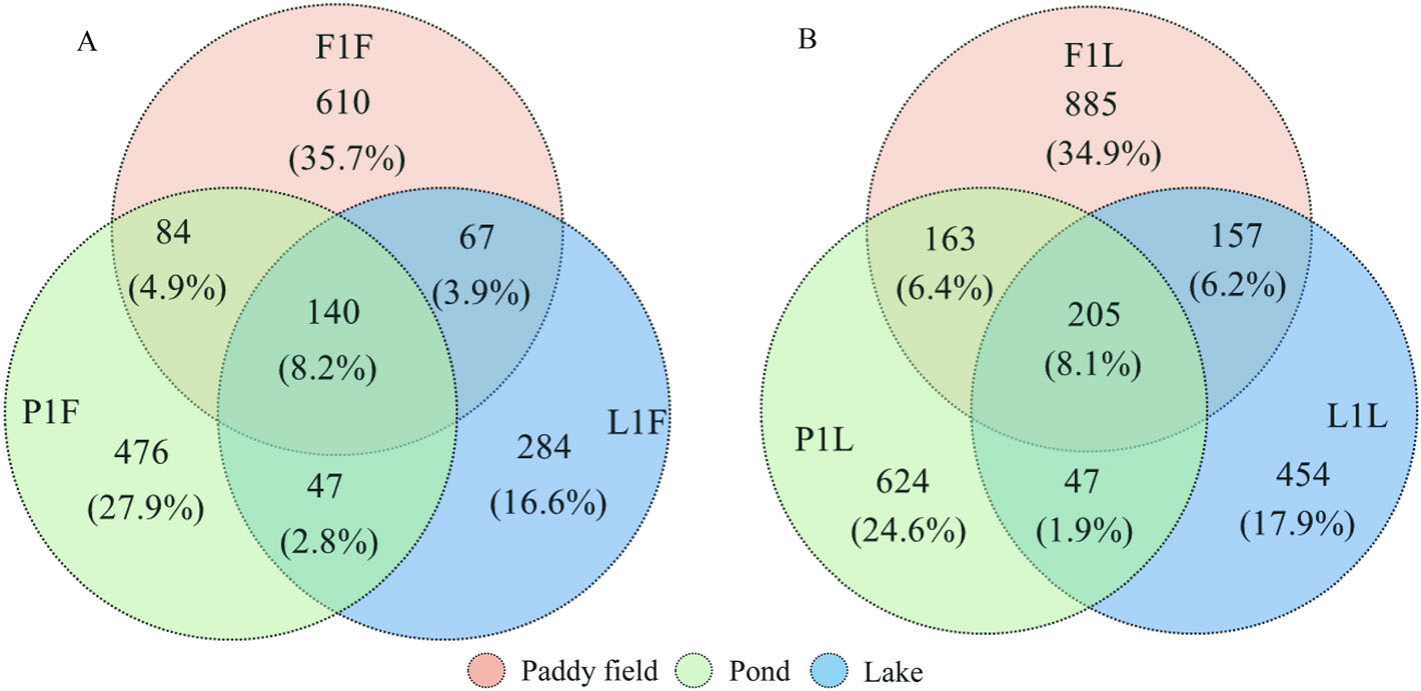
The Venn chart on gut microbial species for three different habitats at 60d, different circles represented different habitats, the percentage indicated number of species/total species in the three habitats, the grouping details were listed in Table 2

The microbial community was relatively complex at 60 d, especially in the foregut. At 60 d, the species of microbes were significantly more abundant in turtles from the fields, followed by those from ponds and lakes. There were 140 common species (8.2 %) in the foreguts of turtles from the three different habitats (Fig. 4a); *Flavobacterium* sp., *Pseudomonas* sp., *Chryseobacterium* sp. and two species belonging to Xanthomonadaceae were relatively abundant. *Cetobacterium somerae* was more abundant in turtles from paddy fields than in those from ponds and lakes. For the hindgut, there were 205 common species (8.1 %) in turtles from the three different habitats (Fig. 4b). Among these, one species belonging to Bacteroidaceae was abundant in all habitats. *Cetobacterium somerae*, *Epulopiscium* sp., *Pseudomonas* sp., *Stenotrophomonas* sp. and *Flavobacterium* sp. were more abundant in turtles from paddy fields and lakes than in ponds, while *Clostridium* sp. and *Epulopiscium* sp. were relatively abundant in specimens from ponds. Moreover, *Chryseobacterium* sp., *Parabacteroides* sp., *Sphingobacterium faecium, Clostridium perfringens*, *Pseudomonas* sp., *Bacteroides* sp. and *Pseudomonas* sp. commonly existed in samples from lakes and paddy fields but did not appear in pond samples. At 120 d, specific foregut microbes were more abundant in pond turtles (74 %) than paddy field turtles (33.4 %), and the common species accounted for 18.6 %; for the hindgut, specific microbes were more abundant in paddy field turtles (44 %) than pond turtles (34.4 %), and the common species accounted for 26.1 % (Fig. S3).

**Fig. 5:**
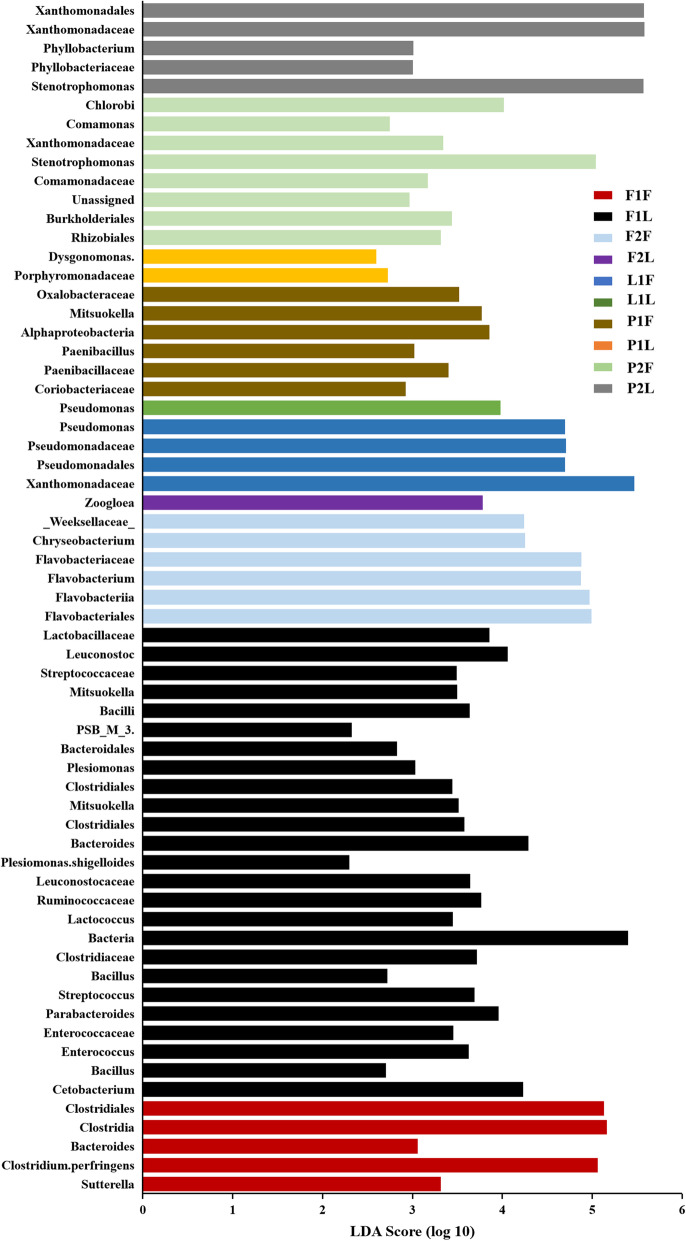
The species with significant differences in abundance for different groups and their effects, the LDA Scores (≥2) were listed and the higher score means bigger effects, the grouping details were listed in Table 2

LEfSe analysis was also conducted to identify representative microbes among various groups. For the initial groups, representative genera were *Weissella*, *Cetobacterium*, *Chryseobacterium*, *Epulopiscium*, *Escherichia*, *Flavobacterium*, *Lactococcus*, *Leuconostoc*, *Plesiomonas*, *Romboutsia*, *Sarcina* and *Stenotrophomonas*. For groups cultured in different habitats, F1L contained more different species than the other groups, including members of *Cetobacterium*, *Lactobacillaceae*, *Bacteroides*, *Parabacteroides*, *Plesiomonas*, and several species belonging to the phylum Firmicutes presented higher LDA scores than those of the other groups. For F1F, the representative taxa were *Sutterella*, *Bacteroides* and *Clostridiales*. For samples from the lake, Xanthomonadaceae and Pseudomonadales were representative taxa, especially at 60 d. The representative microbes in pond turtles were numerous and belonged to various phyla, especially the phylum Proteobacteria, and some unassigned species were found turtles from this habitat (Fig. 5).

### Functional predictions

**Fig. 6:**
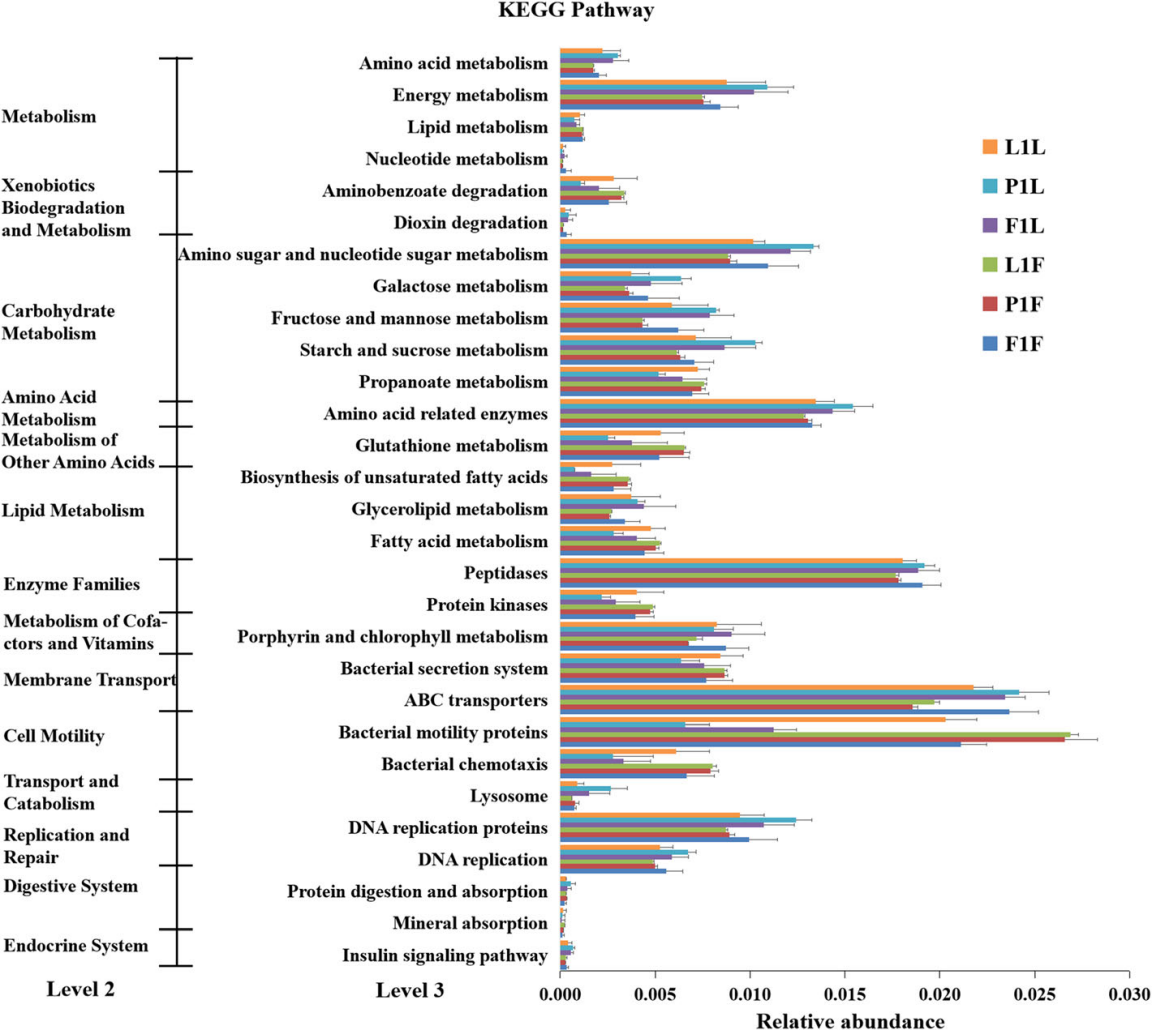
The predicted functional categories and pathway in KEGG level 3, the group details were listed in Table 2

The nearest sequenced taxon index (NSTI) was developed to quantify the availability of nearby genome representatives for groups (Table S2). In total, 39 predicted functional categories that represented 7 pathway maps in level 2 were indicated by PICRUSt, including 275 functions on level 3. Culture period and habitats had significant effects on metabolism such as amino acid and carbohydrate metabolism, environmental and genetic information processing such as membrane transport, replication and repair, especially at 60d (Fig. S4). At 60 d, the functional microbiota in foregut related to amino acid and carbohydrate metabolism was distinct higher in paddy field samples compared to those from ponds and lake, while in hindgut, the functional microbiota were more abundant in pond samples than lake and paddy field (Fig. 6, Fig. S4).

## Discussion

Turtles had the same general microbiota regardless of origin, body size and habitat and presented fast adaptation after allocation to different habitats [28]. The differentiation of growth, behavior and physiology of the homologous turtles appeared under different living habitats in a short period. Environmental changes can substantially influence the intestinal microbiome of mammalian and aquatic animals [29, 30]. The differences might be attributed to living space[6], water quality, food composition and abundance [31, 32], and prey and predation conditions for different habitats [33]. Considering the similarity of natural conditions, such as geographical location, climate, rainfall and temperature, among the mentioned three habitats, the food intake and relative living space might be the main factors determining the growth and physiology of turtles in this study, referring to the researches of aqutaic animals as perch (*Perca fluviatilis*), crucian carp (*Carassius auratus*) and African cichlid fishes [34–36]. Wild turtles are predominantly carnivorous and prey on small fish, mollusks, crustaceans, insects or their larvae, and occasionally some plant seeds, but food abundance is affected by the aquatic environment, competitors and natural enemies in different habitats [37, 38]. In the present study, turtles in paddy fields and ponds were regularly fed artificial feeds, but no such feeds were provided for turtles in the lake during the experiment. In addition, gastropods and insect larvae commonly exist as supplementary food in lakes and paddy fields but rarely exist in ponds [39]. The stocking density in lakes was undoubtedly lower than that in paddy fields and ponds, and the lake environment was relatively stable with capacious water and less disturbance. However, more competitors, predators and parasites existed in the lake, but negligible interspecific competition occurs in this habitat [40]. The paddy field in this study was a complicated habitat with environmental features such as common fields and ponds. The paddy field provided spacious living space, and rice plants served as shelter for turtles. The high growth rate of turtles in this habitat might be attributed to the relatively low stocking density and sufficient food in paddy fields. He et al. (2017) demonstrated that the taste of turtles cultured in paddy fields was better than that of turtles in cultured ponds based on the texture and chewiness of the meat, which might also be due to the broad space of paddy fields for turtle activities [18]. All of these results indicated that the extensive living space of paddy fields could promote growth and quality with proper amounts of food.

Food and feeding strategies obviously affected the morphology and function of the digestive system [41], and a previous study on perch demonstrated that the relative gut length was shorter under stress conditions such as food shortage [34]. For cultured fishes as gilthead sea bream (*Sparus aurata*) and rare minnow (*Gobiocypris rarus*), sufficient feeds might enhance digestive function and promote the development of the gut at an earlier feeding stage, but continuous regular feeding with sufficient food might decrease appetite and digestive activities, along with changes in gut morphology and structure [42, 43]. The gut presented obvious adaptation to habitat, and the relative length of the gut was significantly lower in ponds than in paddy fields and lakes. This might be related to the complicated food composition in lakes and paddy fields, and increased nutrient absorption and prolonged intestinal transit time for turtle [44], which was also found in gibel carp (*Carassius auratus gibelio*) [45]. Although the turtles in ponds were apparently fed to satiation during the experiment, the fixed and simple artificial feed might not be compatible with the ingestion habits of the turtles, and the food species or types also influenced the internal environment and gut microbial communities [46].

The gut microbiota was closely associated with host physiological metabolism, nutrient utilization, nutritional status, immunity, and even health for aquatic animals [47, 48]. The microbes originally derived from previous generations of experimental animals as mouse and human beings in previous studies played important roles in the formation of gut microbial communities and microecological systems [21, 49]. The habitats would also affect the gut microbiota and there were significant differences in gut microbial composition under different habitats for zebrafish (*Danio rerio*), mice and Antarctic seals in previous studies [50, 51]. In general, the microbial population is less diverse in diseased organisms than in healthy organisms. The gut microbial species were more abundant in paddy fields and ponds than in lakes at 60 d, while the species were fewer, and no obvious differences were found among the three habitats at 120 d. This might be due to an obvious reduction in feed intake at 120 d. The dominant phyla and genera were relative similar regardless of the habitats at 120d, and the appearance of dominant phyla and genera was more driven by sampling time than habitats. But the microbial composition were distinct in different habitats at 60d, and both the sampling time and habitats affected the appearance of dominant phyla and genera during the cultivation (Fig. 3). The study on threespine stickleback (*Gasterosteus aculeatus*) showed the composition and abundance of gut microbial communities varied under different habitats to adapt to habitat heterogeneity [52]. In previous studies on zebrafish and dogs, food was deemed as a main factor that influenced gut morphology, homeostasis and microbiota, providing nutrients for the body and acting as a fermentation substrate for gut microbes [53, 54]. The microbial gut communities varied greatly when the Atlantic salmon (*Salmo salar*) were fed diets of different compositions [55]. Therefore, the gut microbita of turtles would be also affected by food supply in various habitats.

Ambient water conditions such as temperature and dietary changes affect the microbiome composition in Atlantic salmon [56, 57], and a suitable diet is conducive to improve the intestinal environment and increase the abundance of probiotics [48]. The PICRUSt functional predictions revealed that both the cultural periods (different seasons) and habitats had significant effects on metabolism, especially amino acid and carbohydrate metabolism, which also indicated the key role of food intake on the gut microbial community in mouse [58]. Moreover, the gut microbiota further influences the metabolic activity of the host as African turquoise killifish (*Nothobranchius furzeri*) [59].

Most previous studies on fish, poultry and mammals have focused on factors that affect the gut microbial community, such as genotype, rearing conditions and diet [60–62]. However, the causality between the microbial community and specific diseases is ambiguous, such as obesity in rodents and humans [63, 64]. Healthy individuals often have intricate and stable gut microbial communities, and pathogenic bacteria might disturb homeostasis and microbial balance, which may manifest as a reduction in gut microbial species and richness. In contrast, in recent studies on grass carp (*Ctenopharynodon idellus*), more bacteria and higher alpha diversity were observed in diseased intestines than healthy intestines, and the richness of bacteria could not fully indicate health status [65]. The representative microbes that could reflect the balance of microbial communities and contribute to intestinal health should be considered, and they might also vary in different species or life stages.

For the turtles in this study, the dominant phyla were Proteobacteria, Bacteroidetes, Firmicutes and Fusobacteria in different habitats, which were similar to the taxa in freshwater fish such as crucian carp, grass carp, and bighead carp (*Hypophthalmichthys nobilis*) [66] and marine turtles such as green turtles (*Chelonia mydas*) [28]. Previous studies indicated that there was a clear difference in composition between aquaculture-reared and wild aquatic animals: in the wild species, Proteobacteria was always the most abundant phylum, whereas Firmicutes was the most abundant phylum in the aquaculture-reared species [67, 68]. For the turtles in this study, it was also found that Firmicutes was the most abundant phylum in the guts of turtles sampled from the greenhouse under the initial intensive aquacultural conditions, whereas Proteobacteria was the most abundant phylum after cultivation in ponds, lakes and paddy fields, especially at 120d. The results also indicated that the gut microbiota of turtles had both intrinsic and distinct environmental characteristics. Aeromonas, Chryseobacterium and Citrobacter commonly exist in European pond turtles kept in breeding centers, and there were obvious differences in bacterial composition and abundance for turtles of different ages [69]. The composition and abundance of gut bacteria also varied with different physical statuses, and the virulence and prevalence of pathogens were suppressed in healthy individuals [70]. Cetobacterium, Cyanobacterium and Clostridiaceae were more abundant in healthy fish, whereas Aeromonas, Vibrio and Shewanella OTUs were more abundant in diseased individuals [71]. *Enterococcus* spp. and *Citrobacter* spp. were the dominant bacteria in healthy turtles, while *Citrobacter* spp., *Aeromonas spp.* and *Bacillus* spp. were predominant in diseased turtles [72]. *Lactococcus garvieae*, *Citrobacter freundii and Edwardsiella tarda* were commonly pathogenic bacteria in aquatic environments [73]. In this study, *Edwardsiella* spp. was occasionally found in samples from ponds but rarely found in those from paddy fields and lakes. *Aeromonas* spp. and *Citrobacter* spp. were absent in almost all samples. *Bacillus* spp. were more abundant in paddy fields than in lakes and ponds at 60 d. *Pseudomonas* spp. existed widely and were rich in most samples except hindgut samples from pond turtles at 60 d. In addition, the nonpathogenic bacteria *Enterococcus faecium*, *Enterococcus hirae*, *Haemophilus segnis*, *Ochrobactrum anthropi* and *Pseudomonas* spp. could also induce carapace and plastron damage when the cultural environment became poor. The relationship between gut microbial communities and bodily health was not static, and the gut microbial community was mutually adapted to the internal and external environments. Therefore, the relationship among microbial communities in the gut, culture water and soil should also be detected to reveal the adaptation of turtles to different habitats.

It is necessary to optimize feeding regimes and cultural conditions to improve the economic and environmental sustainability of aquaculture. Burgeoning culture modes in reconstructive outdoor ponds and paddy fields have been developed to replace hothouse cultivation, especially in the later life stages before coming into the market. In this study, the turtles cultured in paddy fields presented the maximum growth rate. The rice production was relative stable or increased under a low area of furrow or ponds in field paddy (≤ 10 % of the total planting area) and the mutual promotion of rice and aquatic animals. Moreover, coculture could increase the value of rice and turtles with a marked decrease in fertilizer and pesticide utilization. The rice-turtle coculture modes were widely developed and were suitable in both single and double cropping rice cultivation area. All of these results indicated that the coculture mode was economic and ecological. The coculture mode could be optimized by reasonable soil, water and fertilizer management, especially nitrogen fertilization and creating a feeding regime of turtles on the basis of digestibility, which could minimize nutrient outputs and decrease the environmental impacts of intensive culture [74, 75]. Rice-turtle coculture is an economic and ecological integrated culture mode that might play important roles in paddy field environmental protection and food security due to the sharp decrease in the utilization of chemical fertilizers and pesticides with this method compared to that under traditional planting modes. The mutual promotion of the field environment and turtle health were preliminarily detected in the present study, but the effectiveness and potential of this method should be investigated more systematically in future work.

## Conclusions

The juvenile Chinese soft-shelled turtles could adapt to different habitats, including natural lakes, artificial ponds and paddy fields. The divergence in growth, appearance, physiological characteristics and gut microbial communities was observed within a relatively short term. The species of microbes were significantly more diverse in paddy field specimens than in those from ponds and lakes. The diversity and abundance of gut microbes were also higher for turtles from paddy fields than for those from lakes and ponds. Significant divergence was found in summer, whereas relatively less diversity was detected in late autumn. The abundances of dominant phyla and genera were obviously different in various habitats at specific sampling times. Sampling time and habitat had significant effects on turtle metabolism, especially amino acid and carbohydrate metabolism. Rice-turtle coculture is a potential ecological and economic farming mode that plays important roles in wild turtle protection, food security and paddy field environment improvement.

## Methods

### Experimental habitats and turtle rearing

The turtles (*Pelodiscus sinensis*, Japanese strain) were intensively bred in a standardized aquafarm of Xijiang Aquaculture Co., Ltd., located in Anqing, China. The turtles were stocked in cement tanks in hothouses with relatively stable conditions (temperature was 30.0 ± 1.0 °C and water depth was approximately 0.5 m) before being allocated to different experimental habitats. The turtles were fed to apparent satiation once a day with commercial feed containing 46 % crude protein (Haihuang, Hangzhou, China). Thereafter, thousands of juvenile turtles of a similar size of approximately 340 g were purchased and randomly divided into three groups that were allocated to different experimental culture habitats as follows. Natural Lake (L): Bohu Lake is located in Anqing, Anhui Province, China (E116°22′, N30°13′) and belongs to the Yangtze River basin. It covers 217 km^2^, and the average water depth is approximately 3.5 m from July to October. The lake is abundant in fish, shellfish and other aquatic species. Two thousand marked turtles were released to the lake, and no artificial feeds were provided. The artificial release was conducive to the recovery of the wild turtle population. Artificial Pond (P): The quadratic artificial ponds equipped with feeding and basking facilities were located in the above mentioned standard aquafarm (116°54′E, 30°28′N). The experimental ponds were approximately 2000 m^2^ and 1.5 m deep. One thousand turtles were allocated to the pond. The turtles were fed commercial feed that contained 43 % crude protein (Haihuang, Hangzhou, China) twice daily at 09:00 AM and 16:00 PM, and the daily feeding ration was 4 % during the experiment. Paddy Field (F): The experimental paddy fields (E116°21′, N30°18′) were approximately 2000 m^2^ and surrounded by facilities to prevent escape. The area was modified for turtle cultivation with a 200 m^2^ pond (1.5 m deep), which was approximately 10 % of the total field area. Two hundred turtles were allocated to each paddy field. The turtles were fed commercial feed twice a day like those in ponds, but the feeding ration was 3 %. The rearing experiment was conducted for 120 days from July to November. Air temperature was monitored at 11:00 AM every day during the experiment, which varied in the range of 22.5 °C ~ 35.8 °C. Water temperature, pH and dissolved oxygen were monitored daily with a multiparameter water quality analyzer (YSI ProPlus, Yellow Springs, Oh, USA). In addition, ammonium nitrogen and nitrite nitrogen were measured weekly. During the experiment, the water temperature was 21.8 °C ~ 33.6 °C, pH 7.8 ~ 8.2, DO > 5.0 mg/L, ammonium nitrogen < 0.5 mg/L, and nitrite nitrogen < 0.2 mg/L. Partial water changes were performed when the water quality became poor in the ponds and the small ponds in paddy fields. The change interval was approximately 20 days in summer and 30 days in autumn.

### Measurement and sampling

Turtles were randomly collected at 0 d and 60 d, and then as many as possible were collected at 120 d for measurement. The turtles collected from different habitats were randomly numbered, the investigator who selected individuals for analysis was unaware of the grouping details, and another investigator (also unaware of grouping details) conducted the anesthetic and anatomical procedures. Every three male individuals with no trauma, bruises or scars from each habitat and cultural periods were collected for sampling. The turtles were anesthetized and euthanized during measuring and sampling. The turtles were anesthetized after 48 h of fasting by intramuscular injection with tiletamine and zolazepam (1:1) at a dosage of 30 mg/kg. The turtles were under deep anesthesia and unconscious within 15–20 min after injection from the left foreleg. The somatotype index, including body weight, carapace length, carapace width and calipash lateral width, was measured. Then, turtles were quickly decapitated in an unconscious state and dissected by sharp bone shears. The livers, clumpy fat, and guts were carefully removed on ice and weighed under sterile conditions. Gut length, i.e., the length from the end of the esophagus to the end of the rectum were separated and made straight and then measured without tensile force by using an electronic Vernier caliper (Guanglu. Guilin, China). The gastrointestinal tract of turtle is structurally complex and the morphology, digestive function are different in different intestine parts. In consideration of the potential differences on morphology, digestive function and microbial communities for different gut sections, we chose both foregut and hindgut as objects to compare the differences on microbial composition and dynamic variation in different habitats and sampling time. The gastric area (expressed as foregut “F”) and rectum (expressed as hindgut “L”) were separated, rapidly frozen in liquid nitrogen, and then stored at -80 °C until DNA extraction for microbial analysis. The grouping details are listed in Table 2. The specific growth rate (SGR), hepato-somatic index (HSI), fat-somatic index (FSI), and gut-somatic index of weight (DSI_W_) and length (DSI_L_) were calculated. All operations on turtles were conducted in accordance with the institutional animal care guidelines and the supervision of Anhui Academy of Agricultural Sciences committees.

### Bacterial DNA extraction and 16 S rRNA gene amplicon sequencing

Bacterial DNA extraction was conducted using a TIANamp Stool DNA Kit (DP328, TIANGEN, Beijing, China) according to the manufacturer’s instructions. The V4 ~ V5 variable region of the 16 S rRNA gene was amplified by the bacterial primers 515 F (5’-GTGCCAGCMGC CGCGGTAA-3’) and 907R (5’-CCGTCAATTCMTTTRAGT TT-3’) with overhang adapters attached. The PCRs (25 µL) contained approximately 2.5 µL DNA templates (5 ng/µL), 5.0 µL reverse/forward primer (1 µM), and 12.5 µL 2×KAPA HiFi HotStart Ready Mix. PCR was performed on a Step One Plus Real-time PCR System (Thermo Fisher Scientific, Waltham, MA, USA) with the following program: 95 °C for 3 min, followed by 25 cycles of 30 s at 95 °C, 30 s at 55 °C, and 30 s at 72 °C with a postamplification extension of 10 min at 72 °C. The products were confirmed by agarose gel electrophoresis (Peiqing, Shanghai, China). AMPure XP beads (Beckman Coulter, Indianapolis, IN, USA) and fresh 80 % EtOH were used to purify the 16 S V4 and V5 amplicons away from free primers and primer dimer species for index PCR. Dual indices and Illumina sequencing adapters were attached by using the Nextera XT Index Kit (FC-131-1002, Illumina, San Diego, CA, USA). PCR was performed on a thermal cycler using the following program: 95 °C for 3 min, followed by 8 cycles of 30 s at 95 °C, 30 s at 55 °C, and 30 s at 72 °C with a postamplification extension of 5 min at 72 °C. AMPure XP beads were used to clean up the final library before quantification, normalization and pooling. The purified bacterial DNA samples were sent to Sangon Biotech Co., Ltd. (Shanghai, China) for Illumina MiSeq sequencing.

### 16 S Metagenomics sequencing analysis

The sequencing analysis methods were mainly as described by Campos et al. (2018) and Abdelrhman et al. (2016) [28, 76]. The obtained DNA reads were compiled in FastQC version 0.11.5 for further processing. QIIME version 1.9.1 was used for microbiome analysis of raw DNA sequencing data, including demultiplexing and quality filtering, OTU picking, taxonomic assignment, phylogenetic reconstruction, diversity analyses and visualizations. The barcode and primer sequences were cut off after the samples were loaded, read pairs were merged using PANDAseq assembler version 2.10 for raw tags, and the sequences were filtered if there was no overlap between them. Then, the chimeras and host sequences were further filtered for clean tags. Singletons were removed before operational taxonomical unit (OTU) clustering (with an identity threshold of 97 %). The valid data were clustered into OTUs using UPARSE. The rarefaction curves for each sample were produced, and diversity values were estimated on rarefied OTUs [77]. The distances among samples were calculated according to the abundance, and the samples were clustered on OTUs to evaluate the similarity. The cluster dendrogram and a phylogenetic tree were also built. Specific differences in community composition were determined using PCoA based on the Bray-Curtis distance matrix. OTUs were taxonomically classified using USEARCH (a unique sequence analysis tool) version 5.2.236 against GreenGenes databases and compiled into each taxonomic level. The composition, abundance and diversity analyses of OTUs were conducted for the species richness and evenness and mutual or proper traits of OTUs for various samples or groups. A test of the significance of differences in OTU composition was conducted using LEfSe analysis to identify the various species. The prediction of microbial community function was conducted by using PICRUSt to evaluate the abundance of functional genes in the samples [78].

### Statistical analysis

All differences among biometric measurements were determined by analysis of variance (ANOVA) using SPSS 20.0. The measured data were subjected to one-way ANOVA. Differences among treatments were tested by Tukey’s multiple range test, and *p* < 0.05 indicated statistically significant differences. Duncan’s multiple comparison was carried out to determine the difference among repeated groups. All statistics on gut microbiota were conducted by using R (version 3.2.2).

Dr. Benli Wu, Long Huang, Jing Chen and Ye Zhang are research assistants at Fisheries Research Institute, Anhui Academy of Agricultural Sciences, and the research directions involve aquatic animal nutrition and environment, aquatic ecology and aquaculture.

Dr. Jun Wang is a lecturer at State Key Laboratory of Eco-hydraulic in Northwest Arid Region of China, and the research directions were fish ecology and aquatic resource protection.

## Supplementary information

**Additional file 1: Fig. S1.** The cluster dendrogram and phylogenetic tree for samples on OTUs, the grouping details were listed in Table 2.

**Additional file 2: Fig. S2**. The PCoA (principal co-ordinates analysis) on Bray-Curtis including initial samples from greenhouse, the different symbols represented different groups from different habitats and cultural periods, the grouping details were listed in Table 2.

**Additional file 3: Fig. S3.** The mutual and specific gut microbial species for groups from fields and ponds at 120d, the grouping details were listed in Table 2.

**Additional file 4: Fig. S4.** The predicted functional categories and pathway in KEGG level 2, the group details were listed in Table 2.

**Additional file 5: Table S1.** Number of observed total and dominant (˃0.1%) OTUs and counts in groups, the group details were listed in Table 2.

**Additional file 6: Table S2.** The nearest sequenced taxon index (NSTI) for groups from different habitats on OTUs,the group details were listed in Table 2.

## Data Availability

All data generated or analyzed during this study are included in this published article [and its supplementary information files]. Raw sequence data on 16s RNA gene had been submitted to the NCBI Sequence Read Archive (SRA) with the accession number PRJNA639398 (http://trace.ncbi.nlm.nih.gov/Traces/sra/).

## Abbreviations

16S rRNA: 16S ribosomal RNA
OTU: Operational taxonomic unit
PCA: Principal component analysis
ANOVA: Analysis of variance
LEfSe: Linear discriminant analysis coupled with effect size
LDA: Liner discriminant analysis
PICRUSt: Phylogenetic Investigation of Communities by Reconstruction of Unobserved States
