# Supplementary figures and images for: Gut microbiota of homologous Chinese soft-shell turtles (Pelodiscus sinensis) in different habitats
Source: BMC Microbiol. 2021 May 11;21:142. doi: 10.1186/s12866-021-02209-y (PMC8112038; doi:10.1186/s12866-021-02209-y)

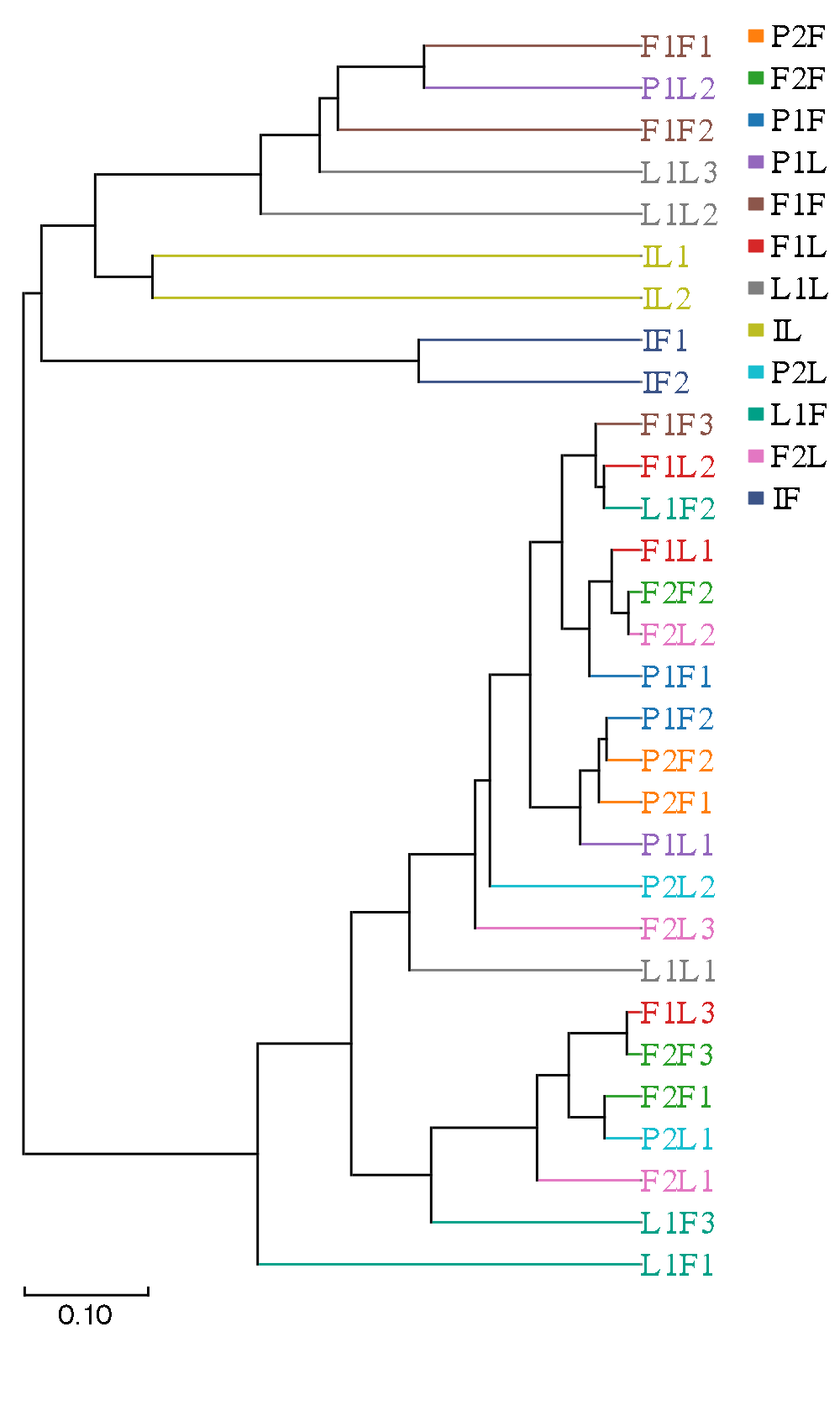

Supplement: Supplementary file 1 — Additional file 1: Fig. S1. The cluster dendrogram and phylogenetic tree for samples on OTUs, the grouping details were listed in Table 2. [file 12866_2021_2209_MOESM1_ESM.tif]

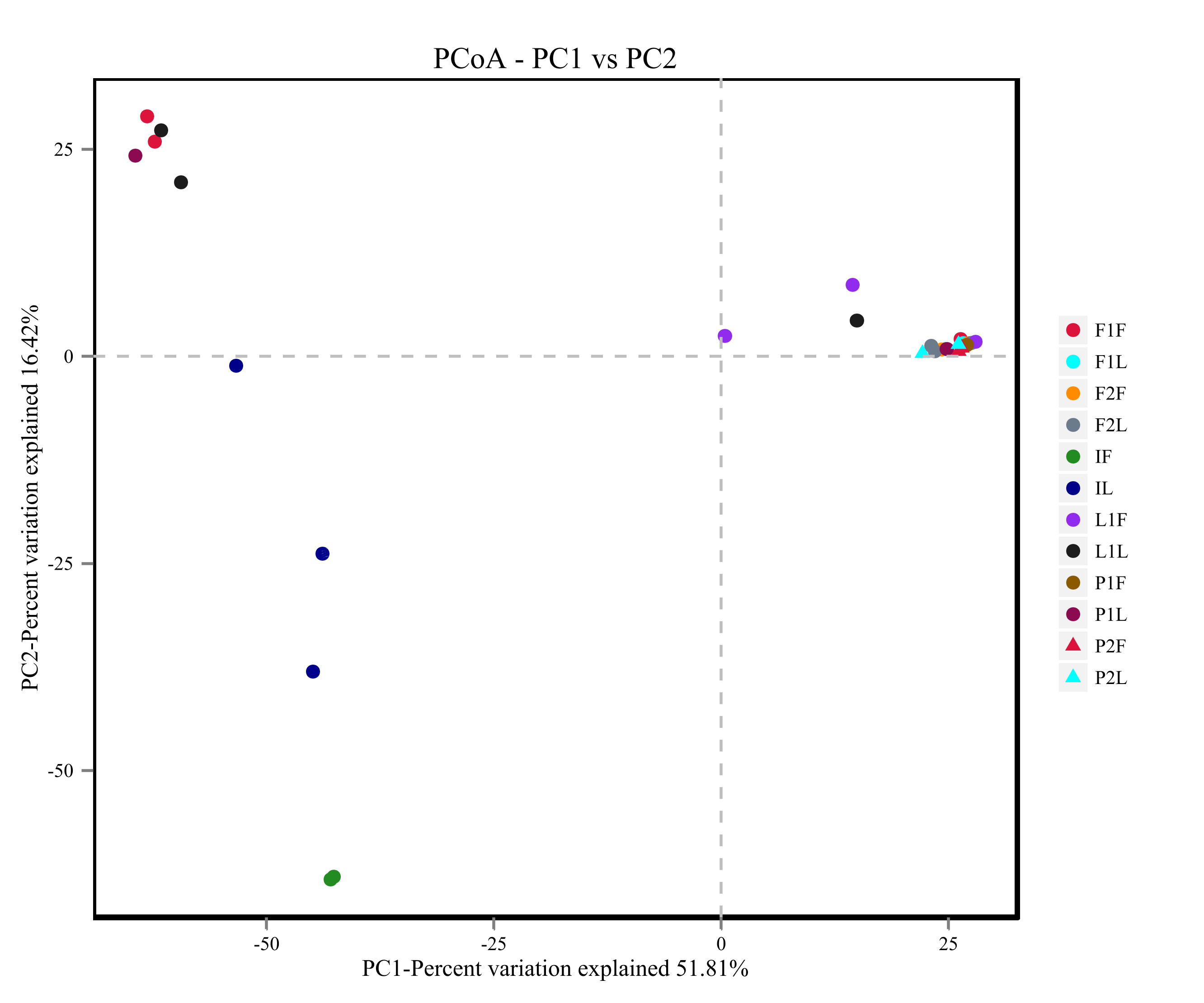

Supplement: Supplementary file 2 — Additional file 2: Fig. S2. The PCoA (principal co-ordinates analysis) on Bray-Curtis including initial samples from greenhouse, the different symbols represented different groups from different habitats and cultural periods, the grouping details were listed in Table 2. [file 12866_2021_2209_MOESM2_ESM.tif]

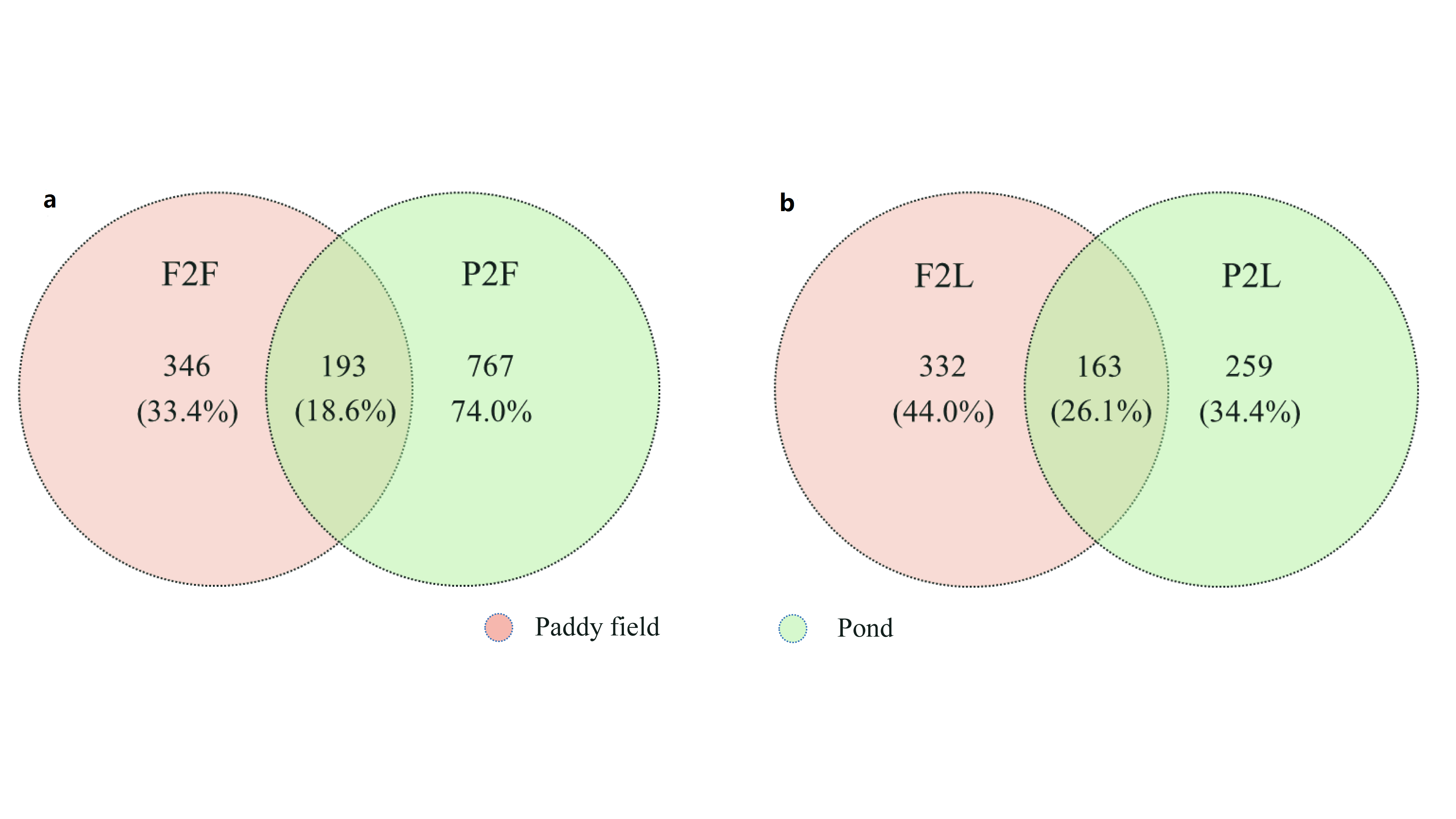

Supplement: Supplementary file 3 — Additional file 3: Fig. S3. The mutual and specific gut microbial species for groups from fields and ponds at 120d, the grouping details were listed in Table 2. [file 12866_2021_2209_MOESM3_ESM.tif]

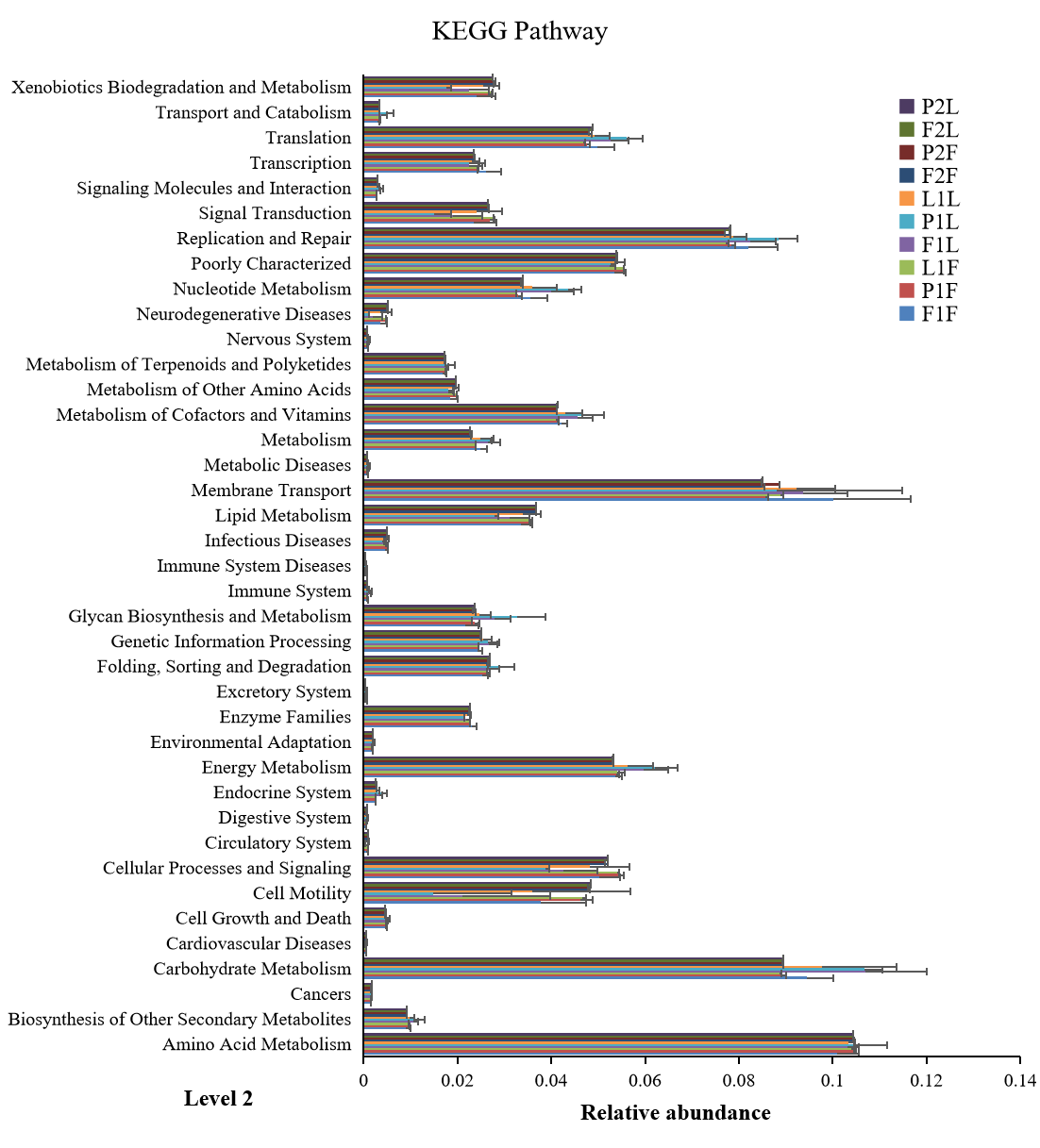

Supplement: Supplementary file 4 — Additional file 4: Fig. S4. The predicted functional categories and pathway in KEGG level 2, the group details were listed in Table 2. [file 12866_2021_2209_MOESM4_ESM.tif]
