# Supplementary material for: Gut microbiota of homologous Chinese soft-shell turtles (Pelodiscus sinensis) in different habitats
Source: BMC Microbiol. 2021 May 11;21:142. doi: 10.1186/s12866-021-02209-y (PMC8112038; doi:10.1186/s12866-021-02209-y)
Supplement: Supplementary file 5 — Additional file 5: Table S1. Number of observed total and dominant (˃0.1%) OTUs and counts in groups, the group details were listed in Table 2. [file 12866_2021_2209_MOESM5_ESM.docx]

**Table S1 Number of observed total and dominant (＞0.1%) OTUs and counts in groups, the group details were listed in Table 2.**

| Groups | Observed OTUs | | Counts | |
| --- | --- | --- | --- | --- |
|  | Total | ＞0.1% | Total | ＞0.1% |
| IF1 | 64 | 17 | 38921 | 39224 |
| IF2 | 72 | 21 | 37587 | 37860 |
| IF3 | 169 | 29 | 35248 | 37816 |
| IL1 | 185 | 48 | 47487 | 48587 |
| IL2 | 118 | 25 | 41255 | 41939 |
| IL3 | 124 | 24 | 44423 | 45051 |
| F1F1 | 306 | 33 | 52320 | 54547 |
| F1F2 | 344 | 36 | 48630 | 51680 |
| F1F3 | 556 | 38 | 40927 | 51163 |
| F1L1 | 822 | 32 | 47875 | 54041 |
| F1L2 | 391 | 37 | 48180 | 51100 |
| F1L3 | 633 | 40 | 40575 | 52107 |
| F2F1 | 245 | 24 | 49956 | 51187 |
| F2F2 | 241 | 24 | 50176 | 51558 |
| F2F3 | 252 | 31 | 52609 | 53774 |
| F2L1 | 256 | 31 | 52798 | 53861 |
| F2L2 | 219 | 29 | 50154 | 52067 |
| F2L3 | 229 | 31 | 51621 | 52498 |
| L1F1 | 249 | 29 | 44833 | 46137 |
| L1F2 | 240 | 27 | 53226 | 54630 |
| L1F3 | 270 | 29 | 50400 | 52082 |
| L1L1 | 479 | 33 | 46428 | 50571 |
| L1L2 | 300 | 39 | 52918 | 54915 |
| L1L3 | 218 | 28 | 48243 | 50002 |
| P1F1 | 232 | 29 | 53538 | 54804 |
| P1F2 | 277 | 34 | 48070 | 51567 |
| P1F3 | 446 | 27 | 47098 | 50734 |
| P1L1 | 481 | 31 | 49044 | 53393 |
| P1L2 | 420 | 27 | 49810 | 51038 |
| P1L3 | 571 | 27 | 50699 | 54762 |
| P2F1 | 805 | 39 | 43424 | 52762 |
| P2F2 | 218 | 19 | 51515 | 52456 |
| P2F3 | 345 | 25 | 51232 | 52526 |
| P2L1 | 295 | 29 | 49189 | 51171 |
| P2L2 | 231 | 31 | 51624 | 52754 |
| P2L3 | 246 | 30 | 51125 | 51985 |
