# Supplementary material for: Gut microbiota of homologous Chinese soft-shell turtles (Pelodiscus sinensis) in different habitats
Source: BMC Microbiol. 2021 May 11;21:142. doi: 10.1186/s12866-021-02209-y (PMC8112038; doi:10.1186/s12866-021-02209-y)
Supplement: Supplementary file 6 — Additional file 6: Table S2. The nearest sequenced taxon index (NSTI) for groups from different habitats on OTUs,the group details were listed in Table 2. [file 12866_2021_2209_MOESM6_ESM.docx]

**Table S2 The nearest sequenced taxon index (NSTI) for groups from different habitats on OTUs,the group details were listed in Table 2.**

| Samples | **NSTI** | | |
| --- | --- | --- | --- |
|  | Total OTUs | ≥2 OTUs | ≥0.1%OTUs |
| F1F1 | 0.1216 | 0.1216 | 0.1241 |
| F1F2 | 0.1207 | 0.1207 | 0.1216 |
| F1F3 | 0.1212 | 0.1212 | 0.1205 |
| F1L1 | 0.1557 | 0.1558 | 0.17 |
| F1L2 | 0.123 | 0.1231 | 0.1242 |
| F1L3 | 0.1367 | 0.1367 | 0.1377 |
| P1F1 | 0.1198 | 0.1198 | 0.1208 |
| P1F2 | 0.1294 | 0.1294 | 0.1311 |
| P1F3 | 0.121 | 0.121 | 0.1226 |
| P1L1 | 0.1362 | 0.1362 | 0.1345 |
| P1L2 | 0.1678 | 0.1678 | 0.168 |
| P1L3 | 0.165 | 0.165 | 0.1655 |
| L1F1 | 0.1279 | 0.1279 | 0.1293 |
| L1F2 | 0.1218 | 0.1218 | 0.1232 |
| L1F3 | 0.1379 | 0.1379 | 0.1376 |
| L1L1 | 0.1309 | 0.1309 | 0.134 |
| L1L2 | 0.0856 | 0.0856 | 0.0868 |
| L1L3 | 0.1327 | 0.1327 | 0.1324 |
| F2F1 | 0.0718 | 0.0718 | 0.0716 |
| F2F2 | 0.073 | 0.073 | 0.0727 |
| F2F3 | 0.0727 | 0.0727 | 0.0727 |
| F2L1 | 0.0724 | 0.0724 | 0.0727 |
| F2L2 | 0.0724 | 0.0724 | 0.0725 |
| F2L3 | 0.0733 | 0.0733 | 0.0735 |
| P2F1 | 0.0882 | 0.0882 | 0.0863 |
| P2F2 | 0.072 | 0.072 | 0.0723 |
| P2F3 | 0.0801 | 0.0801 | 0.0802 |
| P2L1 | 0.073 | 0.073 | 0.073 |
| P2L2 | 0.0724 | 0.0724 | 0.0726 |
| P2L3 | 0.0731 | 0.0729 | 0.0732 |
